# Just Do It: High Intensity Physical Activity Preserves Mental and Physical Health in Elite and Non-elite Athletes During COVID-19

**DOI:** 10.3389/fpsyg.2021.757150

**Published:** 2021-11-10

**Authors:** Nicole Casali, Silvia Cerea, Tatiana Moro, Antonio Paoli, Marta Ghisi

**Affiliations:** ^1^Memory & Learning Lab, Department of General Psychology, University of Padova, Padua, Italy; ^2^Experimental Psychopathology Lab, Department of General Psychology, University of Padova, Padua, Italy; ^3^Nutrition and Exercise Physiology Lab, Department of Biomedical Sciences, University of Padova, Padua, Italy; ^4^Unità Operativa Complessa (UOC) Hospital Psychology, University-Hospital of Padova, Padua, Italy

**Keywords:** lockdown, pandemic (COVID19), physical activity, psychological health, sports

## Abstract

**Background:** The COVID-19 pandemic forced most Italian athletes to cease their regular training activities, with possible consequences on both mental and physical health. The present study aimed at assessing changes in Physical Activity (PA) from pre- to lockdown, and examining the relationships among lockdown PA, quality of life (mental and physical health), motivation to exercise, psychological distress, intolerance of uncertainty, and body dissatisfaction.

**Methods:** A total of 204 athletes of different sports (91 elite; 110 females; mean age = 22.55, SD = 5.76) answered an online survey investigating demographics, sport-related questions, PA (IPAQ-S), quality of life (SF-12), and psychological variables (BREQ-2, DASS-21, IUS-R, and EDI-3-Body dissatisfaction subscale).

**Results:** Both elite and non-elite athletes significantly reduced their PA intensity and volume (*p* < 0.001). Elite athletes did not differ from non-elite in terms of total PA intensity and hours of training during lockdown (*p* > 0.05). Elite and individual athletes showed higher intrinsic motivation to exercise compared to non-elite and team sports (*p* < 0.01). Female athletes displayed higher distress, worse mental health, and higher body dissatisfaction than males (*p* < 0.05). Mediation models showed that vigorous PA positively affected both mental (*p* < 0.05) and physical (*p* < 0.001) health during lockdown, independently of distress and intolerance of uncertainty.

**Conclusion:** The COVID-19 lockdown was taxing for athletes, particularly professionals; those who were able to practice PA at high intensity during lockdown reported better mental and physical health.

## Introduction

Research on the consequences of lockdowns prompted by the COVID-19 pandemic has mainly focused on mental health in the general population and healthcare professionals (see Pappa et al., 2020; Salari et al., 2020 meta-analyses), and identified anxiety, depression, and post-traumatic stress disorder as the symptoms most commonly reported during lockdown, similarly to previous pandemics (Brooks et al., 2020). Relative less attention has been paid to other dimensions of quality of life (e.g., physical health) and specific populations such as athletes (e.g., Håkansson et al., 2020; Mascret, 2020; Mon-López et al., 2020a,b; Pillay et al., 2020; Şenışık et al., 2020; Facer-Childs et al., 2021; Leyton-Román et al., 2021), despite the evidence of a positive association between practicing physical exercise during lockdown and mental health in the general population (Maugeri et al., 2020), college students (Zhang et al., 2020), and healthcare workers (Zhu et al., 2020).

Adopting self-determination theory (SDT; Deci and Ryan, 1985; Ryan and Deci, 2000) as a guiding framework, the present study investigated: (i) the changes in Physical Activity (PA) in Italian elite and non-elite athletes of individual and team sports from pre- to during lockdown, and (ii) the associations among PA, quality of life (mental and physical health), and other relevant psychological variables (i.e., motivation toward PA, general distress, intolerance of uncertainty, and body dissatisfaction) during the first Italian nationwide lockdown.

### The Impact of COVID-19 on Athletes

According to the International Olympic Committee (IOC) consensus statement on mental health in elite athletes (Reardon et al., 2019), sports practice should protect against psychological disorders, given exercise’s antidepressant properties. Interestingly, it has been suggested that when athletes cease to exercise, its antidepressant effect disappears and mood could therefore worsen (Reardon and Factor, 2010). Meta-analytical accounts also support the general benefits of sports practice over health status (Oja et al., 2015). In the COVID-19 context, reports on the athletes’ population consistently showed decreased training activity, unhealthy nutritional and sleep trends during the lockdown, which correlated with poorer mental health (Mon-López et al., 2020a,b; Pillay et al., 2020; Şenışık et al., 2020; Facer-Childs et al., 2021). Less is known about perceived physical health during the pandemic, with only one study investigating individual differences in this variable in a sample of adolescent athletes (McGuine et al., 2021).

Identifying psychological variables related to PA and quality of life (i.e., mental and physical health) during a pandemic is crucial to sustaining athletes at higher risk of experiencing adverse mental and physical consequences under particularly stressful situations.

### Psychological Variables Linked to Quality of Life in Athletes

Self-determination theory (Deci and Ryan, 1985; Ryan and Deci, 2000) identifies three basic psychological needs (i.e., autonomy, competence, and relatedness) that must be satisfied to achieve self-motivation and higher wellbeing. Motivation is defined as the ways in which individuals regulate any of their behavior. More specifically, motivation toward PA is conceptualized as a continuum ranging from amotivation (i.e., lack of intention to engage in PA, non-self-determined regulation) to intrinsic motivation (i.e., practice PA for its own sake, self-determined). Intermediate forms of regulation include external regulation, introjection, identification, and integration (see Markland and Tobin, 2004). Intrinsic motivation toward PA has been associated with a higher commitment to sports practice during COVID-19 lockdown (Leyton-Román et al., 2021) and may be therefore positively related to self-reported PA during the lockdown.

Other factors contributing to understanding athletes’ quality of life under a stressful and uncertain situation such as a pandemic may include general distress, intolerance of uncertainty, and body dissatisfaction (Buckley et al., 2021; Li et al., 2021).

General distress is a state measure of overall negative emotionality, characterized by symptoms of anxiety, depression, and stress (Lovibond and Lovibond, 1995). It has been associated positively with mental and negatively with physical health (Sinclair et al., 2011) and has been recently suggested to be a solid construct in athletes as well (Vaughan et al., 2020).

Intolerance of uncertainty represents a general disposition to interpret uncertain situations as discomforting and has been associated with several psychopathologies, including health anxiety (Gentes and Ruscio, 2011). Under the COVID-19 pandemic, it has been suggested to positively relate to test anxiety in student-athletes (Li et al., 2021).

Body dissatisfaction describes all the concerns with one’s body size and shape and has been indicated as the strongest predictor of eating disorders in athletes (Reardon et al., 2019). Only one study has examined its role in athletes during the pandemic and found higher concerns in female current and former athletes compared to males (Buckley et al., 2021).

### The Present Study

Italy was the first Western country to face the dramatic spread of the virus. For what concerns sports, the course of events was characterized by a high uncertainty and rapidity: on February 23rd, 2020, sports events and gatherings were suspended in the two most affected Italian regions (i.e., Lombardy and Veneto), but it was still possible to train and compete. On March 4th, 2020, sports events and competitions of all levels and disciplines in any public or private locations were suspended in specific areas (the so-called “red zones”). Elsewhere it was still allowed to hold events and competitions, as well as training sessions for elite athletes only, within closed sports facilities or outdoors without spectators; all sports societies and associations had to perform tests aimed at containing contagion among athletes, coaches, managers, etc. At that time, recreational sports and PA could be performed under sanitary recommendations. Starting on March 10th, 2020, these measures were extended to the entire national territory: due to nationwide lockdown measures, all, but vital businesses were closed and leaving one’s house restricted to job/health-related or other very serious reasons. Pertaining to the sport context, sports facilities could be still utilized by athletes of national interest training for the Olympics or other international competitions, behind closed doors; therefore, the only competitions allowed were those organized by international committees and could only be held behind closed doors or outdoors without spectators. On March 24th, 2020, the IOC announced the postponement of the 2020 Olympics to 2021. Then, on April 1st, 2020, suspension of all sports events, competitions, and training sessions was declared on the whole Italian national territory. Meanwhile, Italian federations were issuing their own public statements: the Italian Volleyball Federation (FIPAV) announced the suspension of the B series championship (March 4th); so did the Italian Soccer Federation for all championships (FIGC; March 10th), the Italian Track and field Federation (FIDAL) for all competitions (March 27th), and the Basketball Federation (FIP) for the A series championship (April 7th). Finally, on April 26th, Phase 2 was declared, with the possibility to train for athletes of individual disciplines recognized as being of national interest and for all individuals (team sports included) to train alone.

In this context, the aim of the present study was to investigate changes in PA from pre- to during lockdown, and to examine the relationships between PA, quality of life, and psychological variables (motivation to PA, psychological distress, intolerance of uncertainty, and body dissatisfaction) experienced during the lockdown. More precisely, based on previous literature, we formulated three sets of hypotheses:

(1) Change in PA from pre- to during lockdown

*Hypothesis 1*. There would be a significant decrease in PA intensity from pre- to during lockdown, as previously reported based on retrospective analysis (Mon-López et al., 2020a,b; Pillay et al., 2020; Şenışık et al., 2020; Facer-Childs et al., 2021).

(2) Individual differences in quality of life and psychological variables

*Hypothesis 2a*. Female athletes would report higher intrinsic motivation toward PA, lower mental health, higher psychological distress, higher intolerance of uncertainty, and higher body dissatisfaction compared to males, as previously shown in both non-pandemic (Reardon et al., 2019) and pandemic situations (Mascret, 2020; Mon-López et al., 2020a,b; Pillay et al., 2020; Buckley et al., 2021).
*Hypothesis 2b*. Elite athletes and athletes practicing team sports would display higher intrinsic motivation toward PA compared to non-elite athletes and athletes in individual sports, respectively, as shown in non-pandemic studies (Nielsen and Wikman, 2018; Hendry et al., 2019; Moradi et al., 2020); team athletes would also report lower body dissatisfaction compared to individual ones (Kristjánsdóttir et al., 2019).

(3) Associations between PA during lockdown, quality of life, distress, and intolerance of uncertainty

*Hypothesis 3a*. Vigorous PA reported during lockdown would be positively related to mental health and general distress, as suggested by previous evidence on COVID-19 pandemic (Mon-López et al., 2020a,b; Pillay et al., 2020; Şenışık et al., 2020; Facer-Childs et al., 2021), and possibly also to physical health and intolerance of uncertainty. We focused on Vigorous PA (rather than Walking or Moderate) as it is the most representative of athletes’ regular training activity.
*Hypothesis 3b*. General distress and intolerance of uncertainty would be positively related to both components of quality of life (Lovibond and Lovibond, 1995; Gentes and Ruscio, 2011; Sinclair et al., 2011).
*Hypothesis 3c*. General distress and intolerance of uncertainty would mediate the relationship between Vigorous PA during lockdown and mental health (Model 1).
*Hypothesis 3d*. Mental health and intolerance of uncertainty would mediate the relationship between Vigorous PA during lockdown and physical health (Model 2).

## Materials and Methods

### Participants

*A priori* power analysis was conducted using G^∗^Power (Faul et al., 2007) and the Shiny app MCpowrMed (Schoemann et al., 2017). As for mixed ANOVAs (i.e., considering Time as within factor, Level of sport, Type of sport, and Gender as between factors), a total sample size of 42 was sufficient to obtain a power of 0.80, with a medium *f* effect size (0.25). For 2 × 2 × 2 univariate ANOVAs (considering Level of sport, Type of sport, and Gender) the sample size required equaled 159. Finally, for mediation models, Montecarlo simulations (*n* = 5,000) conducted with MCpowrMed starting from a theory-based covariance matrix with small-to-medium correlations indicated that 174 participants were enough to obtain a power of 0.80 in Model 1 (i.e., testing the effect of Vigorous MET on SF-12 Mental component, with DASS-21 and IUS-R as parallel mediators), while for Model 2 (i.e., testing the effect of Vigorous MET on SF-12 Physical component, with SF-12 Mental component and IUS-R as parallel mediators) the suggested sample size was 140 participants. Therefore, the sample size achieved in the study (*n* = 204) over the timeframe of interest (i.e., Phase 1 of the lockdown, before Phase 2 was announced and measures lessened a bit) resulted satisfactorily. Indeed, participants were 204 athletes (94 males, 91 elite athletes), aged 18–58 (*M* = 22.55, SD = 5.76), practicing different disciplines [93 individual sports (42 track and field, 8 cycling, 7 martial arts, 5 horseback riding, 4 swimming, 4 artistic gymnastics), 111 team sports (44 volleyball, 21 soccer, 11 basketball)]. Participants were considered “elite” athletes if they self-reported competing at the national level or above, and “non-elite” if they competed below the national level. Inclusion criteria were being over 18 of age and practicing a sports discipline at a competitive level. All available data were considered in the analyses, with missing data referring mostly to SF-12 Mental and Physical components (17.15%) and IPAQ-S (26.9% for the total MET score pre-lockdown; 17.6% for the total MET score during lockdown). Table 1 displays the socio-demographic features of the two groups.

**TABLE 1:** Socio-demographic characteristics of elite and non-elite athletes and assessment of their differences according to age, gender, type of sport, BMI, and experience.

	Elite (%)	Non-elite (%)	Test statistic
Age (years)^*a*^	22.82 ± 6.02	22.33 ± 5.55	*t* = −0.61
Gender			χ^2^ = 1.67
Male	47 (51.6)	47 (41.6)	
Female	44 (48.4)	66 (58.4)	
Type of sport			χ^2^ = 11.55***
Individual	54 (59.3)	39 (34.5)	
Team	37 (40.7)	74 (65.5)	
BMI (kg/m^2^)^*a*^	22.35 ± 2.64	22.1 ± 2.86	*t* = −0.65
Experience (months)^*a*^	124.3 ± 90.17	118.18 ± 104.75	*t* = −0.45

*^*a*^Data are reported as mean ± SD.*

***p < 0.001.

*BMI, body mass index.*

### Materials

To investigate participants’ socio-demographic characteristics (i.e., age, gender, self-reported height and weight) and sport variables (i.e., type of sport, experience, number and hours of training pre- and during lockdown) a socio-demographic information schedule was employed.

To measure PA before and during the pandemic, we asked participants to complete the International Physical Activity Questionnaire–Short version (IPAQ-S; Craig et al., 2003; Italian version by Mannocci et al., 2010). This decision stemmed from the need to have a measure of PA that was short, easily implementable online, and validated in Italian. The IPAQ-S is a self-report questionnaire composed of seven items measuring five domains of PA (i.e., job-related, transportation, housework, sport and leisure time, and sitting). PA is expressed in terms of energy expenditure (MET), intensity (walking, moderate, vigorous), and duration (minutes/day, days/week). The IPAQ-S displayed acceptable reliability indices both in the 12-country analysis of the instrument (Spearman’s ρ∼0.80; Craig et al., 2003) and the Italian validation (Cronbach’s α = 0.60; Mannocci et al., 2010). This was the only instrument administered twice.

To investigate motivation, we used the Behavioral Regulation in Exercise Questionnaire-2 (BREQ-2; Markland and Tobin, 2004; Italian version by Costa et al., 2013). This is a self-report questionnaire comprising 19 items investigating four kinds of motivation toward PA: amotivation (e.g., “I don’t see why I should have to exercise”), external (e.g., “I exercise because other people say I should”), introjected (e.g., “I feel guilty when I don’t exercise”), identified (e.g., “I value the benefits of exercise”), and intrinsic (e.g., “I exercise because it’s fun”) on a 5-point Likert scale. The subscales displayed satisfactory internal consistencies in both the original version (α = 0.73–0.86; Markland and Tobin, 2004) and the Italian validation study (α = 0.70–0.87; Costa et al., 2013).

To address psychological distress during the lockdown, the Depression Anxiety Stress Scales-21 (DASS-21; Lovibond and Lovibond, 1995; Italian version by Bottesi et al., 2015) was administered. This is a 21-item self-report questionnaire that investigates symptoms related to depression (e.g., “I could not feel any positive emotion”), anxiety (e.g., “I felt I was having a panic attack”), and stress (e.g., “I felt stressed”) experienced over the past week on a 4-point Likert scale. Findings on the Italian version recommended the use of the total score, assessing “general distress” (α = 0.90; Bottesi et al., 2015), rather than calculating the three subscale scores separately. Therefore, given this suggestion and the purpose of the present study, we focused only on the total score of the DASS-21.

The Intolerance of Uncertainty Scale-Revised (IUS-R; Carleton et al., 2007; Italian version by Bottesi et al., 2019) was used to measure the tendency to interpret uncertain situations as stressful and threatening. This is a self-report questionnaire made of 12 items on a 5-point Likert scale (e.g., “Unforeseen events upset me greatly”). The scale showed good internal consistency, both in the original version (α = 0.91; Carleton et al., 2007) and the Italian validation study (α = 0.90; Bottesi et al., 2019).

The Italian version of the Short Form Health Survey-12 (SF-12; Ware et al., 1996; Italian version by Apolone, 2001) was used to evaluate athletes’ quality of life. This comprises twelve items (e.g., “During the past 4 weeks, have you had any of the following problems with your work or other regular daily activities as a result of your physical health?”); mental and physical health components were derived, as suggested by both the original (Ware et al., 1996) and the Italian validation study (Apolone, 2001) as being reliable.

Lastly, the Body Dissatisfaction subscale of the Eating Disorders Inventory-3 (BD; Garner, 2004; Italian version by Giannini et al., 2008) was used to investigate body dissatisfaction. This contains 10 items that measure the degree of body dissatisfaction (e.g., “I think my stomach is too big”) on a 6-point Likert scale. The EDI-3 questionnaire displayed good internal consistency both in the original version (α = 0.63–0.97; Garner, 2004) and the Italian validation study (α = 0.70–0.92; Giannini et al., 2008).

### Procedure

A convenience sample of athletes was recruited *via* email and personal contacts from April 10th, 2020 (1 month into the nationwide lockdown) to April 29th, 2020 (soon after Phase 2 was announced). Participants accessed a link to the survey implemented on Qualtrics and, after giving their informed consent, filled in the self-reported questionnaires: first, they provided socio-demographic and sport-related information, then answered the IPAQ-S (twice: first, referring to the period before the beginning of the lockdown; then, referring to the past 7 days), and finally the other self-report questionnaires in randomized order. The survey took around 30 min to be completed, and participants did not receive any compensation for their participation. The study was conducted in accordance with the Declaration of Helsinki and approved by the Ethical Committee of the Biomedical Sciences Department of the local university (HEC-DSB/01-20).

### Statistical Analysis

All statistical analyses were performed using RStudio Team (2020). Mixed ANOVAs were used to evaluate pre- to lockdown changes in PA (IPAQ-S scores; number and hours of training per week; Hypothesis 1). Time (pre- vs. lockdown) was considered as the within-subject variable, while Level of sport (elite vs. non-elite), Type of sport (individuals vs. team), and Gender (males vs. females) as between-subjects variables. If a principal effect (Time, Level of sport, Type of sport, and Gender) or an interaction were significant, Tukey’s *post hoc* tests were conducted to inspect those differences.

Then, 2 × 2 × 2 univariate ANOVAs were run to compare elite and non-elite athletes by type of sport and gender in their scores in the self-report questionnaires during lockdown (Hypotheses 2a and 2b). Conventional significance levels were adopted for the DASS-21, the IUS-R, and the BD subscale of the EDI-3 (*p* < 0.05), whereas Bonferroni’s correction for multiple comparisons was applied to the BREQ subscales (*p* < 0.01), SF-12 components (*p* < 0.03), and IPAQ-S intensity of PA (*p* < 0.02) to decrease Type 1 error.

Pearson’s correlations were calculated to first examine the relationships among PA during lockdown and psychological variables (BREQ-2, DASS-21, IUS-R, BD, SF-12). Correlations were interpreted as small when *r* = 0.20–0.39, medium when *r* = 0.40–0.59, and strong when *r* > 0.60 (Zhu, 2012).

Two mediation models (Hypotheses 3a–3d) were tested using the package *psych* (Revelle, 2021). This method allows evaluating the direct effect of a predictor on a dependent variable (c), as well as the direct effect corrected for the effect that one or more mediating variables may have on the dependent variable (*c’*). Moreover, the indirect effect of the predictor (*ab*) can be computed as the effect of the predictor on the mediator (*a*) multiplied by the effect of the mediator on the dependent variable (*b*). All effects can be corrected for one or more covariates (e.g., socio-demographic variables). When the 95% bootstrap confidence intervals for 5,000 resamples do not include the 0, the effect is considered significant. If an indirect effect is not significant, it means that the effect of the predictor is independent of the effect of possible mediators.

## Results

### Pre- to During-Lockdown Changes in Physical Activity According to Level of Sport, Type of Sport, and Gender

The mixed ANOVA for the total MET showed a significant main effect for Time [*F*_(__1_,_301__)_ = 65.80, *p* < 0.001] and Level of sport [*F*_(__1_,_301__)_ = 15.67, *p* < 0.001], as well as a significant Level of sport × Time interaction [*F*_(__1_,_301__)_ = 5.81, *p* = 0.017]. *Post hoc* analyses indicated that there was a significant decrease in total MET from pre- to lockdown (*p* < 0.001) and that elite athletes had higher scores compared to non-elite athletes (*p* < 0.001); scores significantly decreased in both groups and elite athletes had higher total MET scores pre-lockdown only (*p* = 0.001). As for Walking MET, there was a significant main effect for Time [*F*_(__1_,_260__)_ = 55.42, *p* < 0.001] and Type of sport [*F*_(__1_,_260__)_ = 6.85, *p* = 0.009]. *Post hoc* analyses indicated that there was a significant decrease in Walking MET from pre- to lockdown (*p* < 0.001) and that individual sports had higher scores compared to team sports (*p* = 0.011). Concerning Moderate MET, a significant main effect for Time [*F*_(__1_,_308__)_ = 9.56, *p* = 0.002], Level of sport [*F*_(__1_,_308__)_ = 8.52, *p* = 0.004], and Level of sport × Time interaction [*F*_(__1_,_308__)_ = 7.98, *p* = 0.005] emerged. *Post hoc* analyses indicated a significant decrease in scores from pre- to lockdown (*p* = 0.002) and that elite athletes showed higher scores compared to non-elite (*p* = 0.004); the decrease in Moderate MET from pre- to lockdown was significant for elite athletes only (*p* < 0.001) and they had higher scores compared to non-elite pre-lockdown only (*p* = 0.001). With regards to Vigorous MET, results evidenced a significant main effect for Time [*F*_(__1_,_302__)_ = 55.93, *p* < 0.001] and Level of sport [*F*_(__1_,_302__)_ = 17.56, *p* < 0.001]. *Post hoc* analyses indicated a significant decrease in Vigorous MET from pre- to during lockdown (*p* < 0.001) and that elite athletes had higher scores compared to non-elite (*p* < 0.001).

Regarding the number of weekly training, it emerged a significant main effect for Type [*F*_(__1_,_348__)_ = 27.54, *p* < 0.001] and Level of sport [*F*_(__1_,_348__)_ = 41.40, *p* < 0.001], plus a significant Level of sport × Time interaction [*F*_(__1_,_348__)_ = 6.55, *p* = 0.011]. *Post hoc* analyses indicated that, independently of Time, individual athletes did a higher number of trainings per week compared to team athletes (*p* < 0.001). It also emerged that elite athletes did a higher number of trainings per week compared to non-elite only pre-lockdown (*p* < 0.001). Lastly, with respect to weekly hours of training, there were a significant main effect for Time [*F*_(__1_,_392__)_ = 95.07, *p* < 0.001], Level of sport [*F*_(__1_,_392__)_ = 47.30, *p* < 0.001], and Type of sport [*F*_(__1_,_392__)_ = 5.80, *p* = 0.016], together with a significant Level of sport × Time interaction [*F*_(__1_,_392__)_ = 8.10, *p* = 0.005] and Level of sport × Type of sport interaction [*F*_(__1_,_392__)_ = 9.02, *p* = 0.003]. *Post hoc* analyses indicated a significant decrease in the number of hours of training from pre- to during lockdown (*p* < 0.001); moreover, it emerged that elite athletes trained for more hours than non-elite (*p* < 0.001) and individual athletes trained more than team athletes (*p* = 0.020). The decrease in the number of hours of training per week from pre- to lockdown was significant in both elite and non-elite (*p* < 0.001) and elite athletes had higher scores only pre-lockdown (*p* = 0.001); moreover, elite individual athletes trained for more hours per week compared to non-elite individual athletes, and both non-elite and elite team athletes (*ps* < 0.02). Supplementary File 1 displays means and standard deviations for IPAQ measures and training according to Time, Level, Type of sport, and Gender.

### Differences in Psychological Variables According to Level of Sport, Type of Sport, and Gender

The results of the 2 × 2 × 2 ANOVA for the BREQ-2 subscales showed no significant main effects or interactions (*ps* > 0.01) for Amotivation, External regulation, and Introjected regulation subscales. For what concerns Identified regulation, a significant main effect for Type of sport [*F*_(__1_,_193__)_ = 8.08, *p* = 0.005] and Gender [*F*_(__1_,_193__)_ = 7.40, *p* = 0.007] emerged. *Post hoc* analyses indicated that athletes in individual sports displayed higher scores compared to team sports athletes (*p* = 0.006) and that females showed higher scores than males (*p* = 0.008). Results for the Intrinsic regulation subscale revealed a significant main effect for Level [*F*_(__1_,_194__)_ = 12.23, *p* = 0.001] and Type of sport [*F*_(__1_,_194__)_ = 18.48, *p* < 0.001]. *Post hoc* analyses indicated that elite athletes had higher scores compared to non-elite (*p* = 0.008), and that athletes of individual sports displayed higher levels compared to team sports athletes (*p* < 0.001).

Results for the DASS-21 total score indicated a main significant effect for Gender [*F*_(__1_,_196__)_ = 12.72, *p* < 0.001]: females showed higher scores than males in general distress (*p* = 0.001).

As for the IUS-R, there was a significant Level × Type of sport interaction [*F*_(__1_,_196__)_ = 4.52, *p* = 0.035], but *post hoc* analyses showed no significant differences among elite and non-elite, individual and team athletes.

Results for the SF-12 showed no significant main or interaction effects for the Physical component (*ps* > 0.03), while for the Mental component a main significant effect for Gender [*F*_(__1_,_161__)_ = 12.72, *p* < 0.001] emerged; at *post hoc* it emerged that females showed lower scores, indicating poorer self-reported mental health than males (*p* = 0.013).

Lastly, with regards to BD, there were main significant effects for Type of sport [*F*_(__1_,_196__)_ = 4.62, *p* = 0.033] and for Gender [*F*_(__1_,_196__)_ = 38.06, *p* < 0.001]; at *post hoc* it emerged that team sports displayed higher scores, indicating higher body dissatisfaction, than individual sports (*p* = 0.039) and that females showed higher scores than males (*p* < 0.001).

Table 2 displays means and standard deviations for all the psychological variables in the entire sample, elite, and non-elite athletes. Supplementary File 2 displays means and standard deviations for all the psychological variables according to Time, Level, Type of sport, and Gender.

**TABLE 2:** Means and standard deviations of psychological variables in the entire sample, in elite and non-elite athletes.

	Entire sample	Elite	Non-elite
Amotivation	0.68 ± 1.46	0.71 ± 1.45	0.64 ± 1.47
External regulation	1.12 ± 2.07	1.01 ± 1.72	1.21 ± 2.32
Introjected regulation	5.68 ± 2.82	5.79 ± 2.86	5.59 ± 2.79
Identified regulation	13.31 ± 2.39	13.62 ± 2.19	13.05 ± 2.51
Intrinsic regulation	13.31 ± 2.70	14 ± 2.10	12.75 ± 3.00
DASS-21 total score	16.03 ± 10.65	15.97 ± 10.88	16.09 ± 10.51
IUS-R total score	28.74 ± 7.87	29.15 ± 7.43	28.4 ± 8.23
BD total score	29.54 ± 10.72	29.59 ± 11.14	29.5 ± 10.41
Physical component	55.32 ± 5.30	55.37 ± 6.03	55.28 ± 4.60
Mental component	39.59 ± 11.18	40.4 ± 11.70	38.87 ± 10.72

*BD, body dissatisfaction; DASS-21, Depression Anxiety Stress Scales-21; IUS-R, Intolerance of Uncertainty Scale-Revised.*

*Data are mean ± SD.*

### Correlations Between Lockdown Physical Activity and Psychological Variables

The results of the correlation analyses among all the variables showed mostly small correlations between during lockdown PA, quality of life, and psychological measures, as well as small-to-medium correlations between psychological self-report measures. For example, total MET scores during lockdown showed a small positive correlation with intrinsic motivation toward PA (*r* = 0.28), and Vigorous MET scores during lockdown displayed small positive correlations with intrinsic motivation toward PA (*r* = 0.33), mental (*r* = 0.20), and physical health (*r* = 0.21). Furthermore, intolerance of uncertainty and psychological distress were weakly (*r* = −0.22), and strongly (*r* = −0.66) negatively correlated with mental health, respectively. Table 3 displays all the correlations among MET scores during lockdown and the other self-report questionnaires (BREQ-2, DASS-21, IUS-R, SF-12, BD).

**TABLE 3:** Correlations among MET scores during lockdown and self-report questionnaires assessing motivation to PA, psychological distress, intolerance of uncertainty, quality of life, and body dissatisfaction.

	1.	2.	3.	4.	5.	6.	7.	8.	9.	10.	11.	12.	13.	14.	15.
**1. Total MET**															
*2. Walking MET*	0.29														
*3. Moderate MET*	0.77	0.06													
*4. Vigorous MET*	0.89	0.08	0.46												
**5. # trainings/Week**	**0.55**	0.10	**0.35**	**0.56**											
**6. # hours of training/Week**	**0.51**	0.11	**0.25**	**0.56**	**0.69**										
**7. Amotivation**	–0.13	–0.14	–0.08	–0.09	–0.12	–0.21									
**8. External regulation**	–0.08	–0.01	–0.03	–0.10	–0.03	–0.01	0.23								
**9. Introjected regulation**	0.06	–0.05	0.09	0.05	0.10	0.04	–0.13	0.09							
**10. Identified regulation**	**0.23**	0.05	0.10	**0.27**	**0.36**	**0.26**	−**0.18**	−**0.27**	**0.45**						
**11. Intrinsic regulation**	**0.28**	0.10	0.10	**0.33**	**0.28**	**0.30**	−**0.19**	−**0.30**	0.17	0.73					
**12. DASS-21 total score**	–0.08	0.04	–0.00	–0.13	–0.02	–0.13	–0.01	0.06	**0.25**	0.13	–0.03				
**13. IUS-R total score**	–0.15	–0.07	–0.11	–0.13	–0.05	–0.15	0.03	0.14	**0.26**	0.11	–0.09	**0.40**			
**14. Physical component**	0.15	0.04	0.00	**0.20**	–0.12	–0.06	–0.10	–0.15	0.02	0.01	0.12	–0.16	–0.18		
**15. Mental component**	0.16	–0.06	0.08	**0.21**	0.17	0.17	0.08	0.07	−**0.24**	–0.10	–0.02	−**0.66**	−**0.22**	–0.13	
**16. BD total score**	0.08	–0.07	**0.23**	–0.02	0.05	–0.04	–0.04	0.19	**0.43**	0.03	–0.11	**0.26**	**0.24**	–0.13	–0.14

*All | *rs*| > 0.20 are significant for *p* < 0.05, | *rs*| > 0.22 are significant for *p* < 0.01, and | *rs*| > 0.26 are significant for *p* < 0.001. The main correlations are in bold.*

*BD, body dissatisfaction; DASS-21, Depression Anxiety Stress Scales-21; IUS-R, Intolerance of Uncertainty Scale-Revised; MET, Metabolic Equivalent Task.*

### Associations Between Vigorous Physical Activity, Distress, Intolerance of Uncertainty, and Mental Health

To test Hypotheses 3c and 3d, we assessed two mediation models, one in which DASS-21 and IUS-R mediated the relationship between Vigorous MET and SF-12 Mental component (Model 1), and the other in which SF-12 Mental component and IUS-R mediated the relationship between Vigorous MET and SF-12 Physical component (Model 2). In both models, Level, Type of sport, and Gender were included as covariates.

**FIGURE 1:**
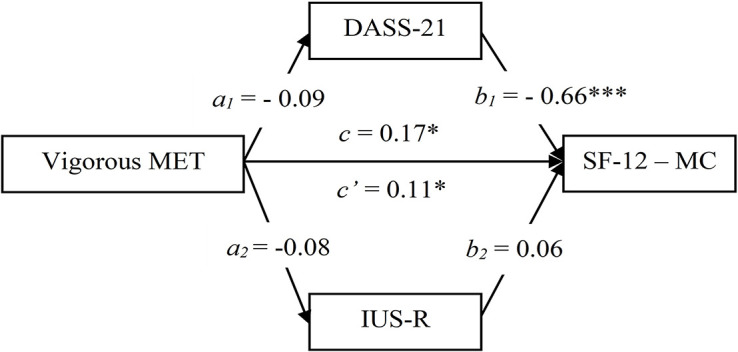
Mediation model testing DASS-21 and IUS-R as mediators of the relationship between Vigorous MET and SF-12 Mental component (M1). ^∗^*p* < 0.05; ^∗∗∗^*p* < 0.001. DASS, Depression Anxiety Stress Scale; IUS, Intolerance of Uncertainty Scale; MET, Metabolic Equivalent Task; SF-12-MC, Short Form Health Survey–Mental component.

Results of Model 1 (Figure 1) showed a significant direct effect (*c’* = 0.11, *p* = 0.035) of Vigorous MET on SF-12 Mental component, removing the effects of DASS-21 and IUS-R; the indirect effect was not significant (*ab* = 0.06, CI [−0.04, 0.15]) for neither DASS-21 (*a_1_b_1_* = 0.06, CI [−0.04, 0.16]) nor IUS-R (*a_2_b_2_* = 0, CI [−0.04, 0.15]).

### Associations Between Vigorous Physical Activity, Mental Health, Intolerance of Uncertainty, and Physical Health

**FIGURE 2:**
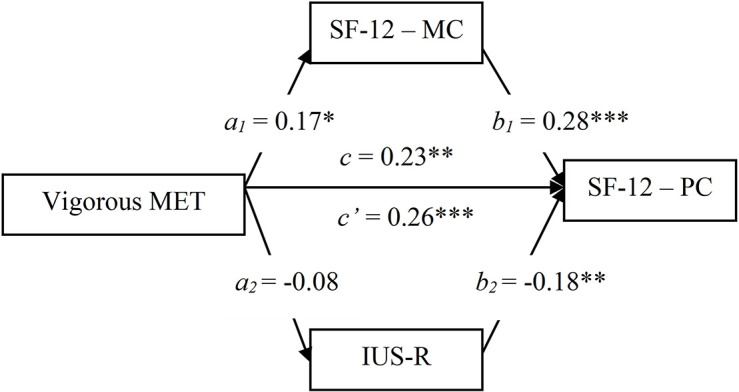
Mediation model testing SF-12 Mental component and IUS-R as mediators of the relationship between Vigorous MET and SF-12 Physical component (M2). ^∗^*p* < 0.05; ^∗∗^*p* < 0.01; ^∗∗∗^*p* < 0.001. BD, body dissatisfaction; DASS, Depression Anxiety Stress Scale; IUS, Intolerance of Uncertainty Scale; MET, Metabolic Equivalent Task; SF-12-PC, Short Form Health Survey–Physical component.

Results of Model 2 (Figure 2) revealed a significant direct effect (*c’* = 0.26, *p* < 0.001) of Vigorous MET on SF-12 Physical component, removing the effects of SF-12 Mental component and IUS-R; the indirect effect was not significant (*ab* = −0.03, CI [−0.11, 0.02]) for neither SF-12 Mental component (*a_1_b_1_* = 0.06, CI [−0.04, 0.16]) nor IUS-R (*a_2_b_2_* = 0, CI [−0.04, 0.15]).

## Discussion

The present study investigated: (i) the impact of COVID-19 lockdown on PA habits in elite and non-elite athletes of individual and team sports, (ii) the effect of gender, level, and type of sport on quality of life and other important psychological variables during lockdown (i.e., motivation to PA, psychological distress, intolerance of uncertainty, and body dissatisfaction), and (iii) the associations among PA and these psychological variables during lockdown.

Concerning the first aim, results confirmed Hypothesis 1 and previous literature (e.g., Pillay et al., 2020; Facer-Childs et al., 2021; McGuine et al., 2021) as they evidenced a significant decrease in PA intensity (i.e., total MET, Walking MET, and Vigorous MET) in both elite and non-elite athletes; Moderate MET were significantly reduced in elite athletes only. Elite athletes displayed higher levels of the total and Moderate MET compared to non-elite athletes pre-lockdown, but these differences were no longer significant during lockdown. For what concerns Vigorous MET, elite athletes showed higher levels compared to non-elite, irrespective of time. In other words, it seems that elite athletes may have felt particularly disadvantaged during lockdown possibly due to the lack of specific facilities and equipment required in their training, and therefore reduced their PA intensity, as previously suggested (Bowes et al., 2020). Moreover, even though IPAQ-S contains examples of Moderate and Vigorous PA, it may be the case that elite and non-elite athletes held different interpretations of what these two levels of PA intensity meant, thus also explaining why elite athletes reported more marked decreases in Moderate PA. Furthermore, athletes in individual sports showed higher levels of Walking MET than team sports, independently of time. This might indicate that those practicing individual disciplines tried and kept more active in their spare time, i.e., outside of their training routine, compared to athletes in team sports.

The number of training sessions per week did not change from pre- to lockdown, while the number of hours of training per week decreased, with elite individual athletes training more compared to their non-elite counterparts, and both elite and non-elite team athletes. These findings are only partly in line with Mon-López et al. (2020a, b) studies on football and handball players during lockdown: in the former, days of training decreased only in males, while hours of training decreased in both males and females, and elite players showed higher training volume (i.e., more days and hours of training per week) during lockdown compared to non-elite. In the case of handball players, the decrease was significant in both men and women, as well as for both days and hours of training; additionally, elite players demonstrated higher training volume than non-elite players both before and during lockdown. These differences could be because both individual and team disciplines were considered in the present study, and in our case individual athletes displayed higher training volumes compared to team ones.

As for the differences in quality of life and other psychological variables during the lockdown, results highlighted a significant effect of the type of sport on identified and intrinsic motivation toward PA, with individual athletes displaying higher levels of self-determined motivation compared to team athletes, in accordance with Hypothesis 2b and previous literature studies (Nielsen and Wikman, 2018; Moradi et al., 2020). Moreover, female athletes showed greater identified motivation compared to males, and elite athletes reported higher intrinsic motivation than non-elite ones, in line with Hypotheses 2a and 2b. Taken together, these findings are in line with literature studies about motivation toward PA not related to pandemic situations (e.g., Hendry et al., 2019) as well as with studies conducted during the pandemic (Leyton-Román et al., 2021). Furthermore, female athletes reported higher levels of psychological distress, body dissatisfaction, and worse mental health compared to males. These findings support Hypothesis 2a and agree with literature studies highlighting that female athletes report more depressive and anxiety symptoms than males (Mascret, 2020; Mon-López et al., 2020a,b; Pillay et al., 2020; Buckley et al., 2021). Contrary to our expectations, team players also seemed to experience greater body dissatisfaction than individual ones. This result could be explained by the fact that our findings showed that the number of hours of training per week decreased, with elite individual athletes training more than both elite and non-elite team athletes. Therefore, even if body dissatisfaction is usually more prevalent in individual sports (Kristjánsdóttir et al., 2019), in this particular case, the pattern seems to be the opposite.

Correlational analyses pointed to small relations between lockdown PA and psychological variables. These results preliminarily indicated that keeping active during lockdown was positively associated with intrinsic motivation to exercise, and quality of life’s physical and mental components, in line with our Hypotheses 3a and 3b and with previous reports of the positive correlations between PA and psychological measures during lockdown (Mon-López et al., 2020a,b; Pillay et al., 2020; Şenışık et al., 2020; Facer-Childs et al., 2021). Additionally, we found significant correlations between physical and mental health, general distress, and intolerance of uncertainty, as previously reported in the general population in non-pandemic situations (Gentes and Ruscio, 2011; Sinclair et al., 2011).

The results of the mediation models further clarified these relationships. More specifically, Vigorous MET and general distress emerged as significant predictors of mental health (Model 1). Moreover, Vigorous MET, mental health, and intolerance of uncertainty significantly predicted physical health (Model 2). Interestingly, the effect of Vigorous PA on both mental and physical health was independent of distress and intolerance of uncertainty, contrary to our expectations (Hypotheses 3c and 3d). These results point to significant positive relationships between Vigorous PA, and physical and mental health during the lockdown, together with significant negative associations between distress and mental health and between intolerance of uncertainty and physical health. In other words, athletes who managed to keep intensely active during quarantine seem to have experienced better quality of life, on both mental and physical levels, possibly due to exercise antidepressant (Cooney et al., 2013) and anxiolytic (Reardon et al., 2021) effects, as well as its general health benefits (Oja et al., 2015). Interestingly, these positive effects appeared to be independent of the psychological distress and intolerance of uncertainty experienced during the lockdown, suggesting that practicing vigorous PA has specific benefits for quality of life in the context of a pandemic.

The present study has some limitations. First, the cross-sectional nature of the study prevents us from making any causal inferences about the directionality of the relationships. Future studies could adopt a longitudinal design to better model the influence of PA on mental health under stressful conditions. Also, since participants answered to the IPAQ-S retrospectively, data on pre-lockdown PA may be subject to recall bias and should be taken with caution. On the same note, the use of IPAQ-S has been criticized as leading to inflated PA rates (Lee et al., 2011). Future online studies should use more reliable instruments to assess PA in athletes. Similarly, response accuracy may be undermined by the length of the survey, even though we adopted randomization to mitigate this possibility.

## Conclusion

The COVID-19 pandemic represents a particularly stressful situation to which most athletes were forced to adapt. Motivation toward PA and quality of life are important parameters for athletes, especially for those that compete at high levels. The strict isolation induced by the COVID-19 pandemic may have caused stress and amotivation, increasing the risk of anxiety and depression. The present study found that elite athletes have reduced their training schedule more than non-elite pairs, indicating that they may have been more badly affected by restrictions compared to non-professional athletes and could require more attention and caution in case of future similarly stressful situations. Level and type of sport differences emerged in motivation to exercise, with elite and individual athletes showing higher intrinsic motivation. Furthermore, our results pointed to gender differences in terms of distress, mental health, and body dissatisfaction experienced during lockdown, with female athletes being more impaired than males in these areas. Finally, mediation models showed that vigorous PA positively affected both mental and physical health during the lockdown.

Taken together, these findings suggest that while COVID-19 lockdown was taxing for athletes, particularly professionals, those who were able to practice PA at high intensity were able to exploit exercise benefits during a stressful situation such as COVID-19 lockdown, reporting better mental and physical health. Consequently, in the case of particularly stressful situations, these results may guide researchers and practitioners in supporting those athletes who may be at increased risk for poor adaptation and lower quality of life.

## Data Availability Statement

The raw data supporting the conclusions of this article will be made available by the authors, without undue reservation.

## Ethics Statement

The studies involving human participants were reviewed and approved by the DSB Human Ethical Committee. The patients/participants provided their written informed consent to participate in this study.

## Author Contributions

NC, SC, TM, AP, and MG: conceptualization, investigation, methodology, and writing—review and editing. All authors have read and agreed to the published version of the manuscript.

## Conflict of Interest

The authors declare that the research was conducted in the absence of any commercial or financial relationships that could be construed as a potential conflict of interest.

## Publisher’s Note

All claims expressed in this article are solely those of the authors and do not necessarily represent those of their affiliated organizations, or those of the publisher, the editors and the reviewers. Any product that may be evaluated in this article, or claim that may be made by its manufacturer, is not guaranteed or endorsed by the publisher.
