# Supplementary material for: Just Do It: High Intensity Physical Activity Preserves Mental and Physical Health in Elite and Non-elite Athletes During COVID-19
Source: Front Psychol. 2021 Nov 10;12:757150. doi: 10.3389/fpsyg.2021.757150 (PMC8631504; doi:10.3389/fpsyg.2021.757150)

Supplementary File 1. Means and standard deviations for PA according to Time, Level, Type of sport, and Gender.

|  |  | Elite | | | | | | Non-elite | | | | | |  |  |
| --- | --- | --- | --- | --- | --- | --- | --- | --- | --- | --- | --- | --- | --- | --- | --- |
|  |  | Individual | | Team | | Total | | Individual | | Team | | Total | | Total (gender) | |
|  | Time | M | F | M | F | M | F | M | F | M | F | M | F | M | F |
| **Total MET** | *Pre-lockdown* | 8046.76± 5702.12 | 7201.81 ± 3764.05 | 6495.4 ± 3063.34 | 7866.36 ± 5174.6 | 7400.36 ± 4789.03 | 7437.62 ± 4243.01 | 4288.64 ± 1896.5 | 5625.07 ± 2847.57 | 4752.61 ± 2158.63 | 5310.92 ± 2848.56 | 4621.74 ± 2074.14 | 5457.04 ± 2818.45 | 5955.48 ± 3874.25 | 6286.74 ± 3596.78 |
|  | *Lockdown* | 2973.65 ± 2646.03 | 4676.94 ± 3185.13 | 3299.47 ± 2123.57 | 3117.15 ± 2304.86 | 3097.77 ± 2438.17 | 4080.55 ± 2944.87 | 2247.23 ± 1322.44 | 2741.92 ± 2567.32 | 2589.47 ± 2080.47 | 3836.06 ± 4177.3 | 2499.83 ± 1901.8 | 3310.87 ± 3507.37 | 2798.8 ± 2194 | 3622.41 ± 3294.67 |
| **Walking MET** | *Pre-lockdown* | 1071 ± 1040.86 | 1042.66 ± 940.38 | 662.91 ± 965.62 | 992.2 ± 862.92 | 893.12 ± 1016.54 | 1022.74 ± 898.99 | 1042.25 ± 803.23 | 1479.98 ± 1294.4 | 822.72 ± 668.44 | 1160.35 ± 1161.39 | 886.98 ± 707.4 | 1301.72 ± 1220.24 | 889.97 ± 866.28 | 1183.93 ± 1099.28 |
|  | *Lockdown* | 399.81 ± 678.81 | 416.07 ± 589.42 | 177.83 ± 270.68 | 317.9 ± 442.32 | 309 ± 555.93 | 378.32 ± 533.57 | 180.12 ± 277.03 | 642.18 ± 1798.64 | 225.66 ± 546.97 | 210.24 ±  419.83 | 213.78 ± 488.43 | 403.07 ± 1246.91 | 260.33 ± 521.76 | 392.91 ± 1012.4 |
| **Moderate MET** | *Pre-lockdown* | 1879.26 ± 2282.36 | 1619.09 ± 1994.26 | 1490.67 ± 1082.27 | 1580 ± 2643.88 | 1740.48 ± 1933.61 | 1605.29 ± 2204.83 | 485.45 ± 465.11 | 981.74 ± 1670.02 | 768 ± 864.16 | 751.11 ± 733.41 | 692.2 ± 782.02 | 857.2 ± 1245.43 | 1222.65 ± 1563.9 | 1160 ± 1727.7 |
|  | *Lockdown* | 545.71 ± 649.94 | 848.7 ± 978.66 | 882.35 ± 1048.45 | 751.43 ± 905.66 | 672.89 ± 828.36 | 811.89 ± 940.1 | 285.45 ± 339.66 | 392.5 ± 491.13 | 714.38 ± 1074.44 | 1086.45 ± 2267.69 | 604.65 ± 956.77 | 783.64 ± 1755.07 | 639.55 ± 888.89 | 795 ± 1475.7 |
| **Vigorous MET** | *Pre-lockdown* | 4724.44 ± 3100.42 | 4789 ± 2622.3 | 4482.11 ± 2422.72 | 5204.71 ± 4005.24 | 4624.35 ± 2813.6 | 4961.37 ± 3227.03 | 2760 ± 2007.33 | 3035 ± 2104.68 | 3261.82 ± 1627.42 | 3626.67 ± 3067.68 | 3128 ± 1727.4 | 3377.54 ± 2698.84 | 4040.16 ± 3019.28 | 3884.4 ± 2445.99 |
|  | *Lockdown* | 2292.86 ± 2036.64 | 3252.17 ± 2323.55 | 2030.32 ± 1441.22 | 2018.82 ± 1340.9 | 2186.72 ± 1806.76 | 2728 ± 2040.7 | 1634.67 ± 1584.87 | 1938.46 ± 1678.45 | 1851.76 ± 1784.88 | 2338.29 ± 1956.33 | 1795.13 ± 1720.33 | 2167.87 ± 1839.11 | 2389.7 1931.15 | 1993.03 1765.94 |
| **# trainings/week** | *Pre-lockdown* | 5.5 ± 2.03 | 5.88 ± 2.3 | 4.21 ± 1.18 | 4.39 ± 1.33 | 4.98± 1.84 | 5.27 ± 2.08 | 4.42 ± 1.44 | 4.07 ± 1.77 | 3.06 ± 1.16 | 3.21 ± 1.13 | 3.4 ± 1.36 | 3.56 ± 1.48 | 4.19 ± 1.79 | 4.25 ± 1.93 |
|  | *Lockdown* | 4.71 ± 1.98 | 5.69 2.85 | 4.47 1.58 | 4.11 ± 1.45 | 4.62 ± 1.81 | 5.05 ± 2.49 | 4.67 ± 1.67 | 4.48 1.7 | 3.4 ± 2.32 | 4.36 ± 1.74 | 3.72 ± 2.22 | 4.41 ± 1.71 | 4.17 ± 2.07 | 4.66 ± 2.07 |
| **# hours of training/ week** | *Pre-lockdown* | 13.66 ± 7 | 13.96 ± 7.99 | 11.11 ± 4.33 | 11.22 ± 5.05 | 12.63 ± 6.14 | 12.84 ± 7 | 8.29 ± 4.82 | 8.04 ± 3.2 | 8.2 ± 2.63 | 9.05 ± 3.52 | 8.22 ± 3.27 | 8.64 ± 3.4 | 10.43 ± 5.37 | 10.32 ± 5.53 |
|  | *Lockdown* | 6.99 ± 4.07 | 9.19 ± 5.37 | 5.94 ± 2.73 | 5.44 ± 2.55 | 6.57± 3.59 | 7.66 ± 4.77 | 5.25 ± 3.51 | 5.63 ± 2.83 | 4.74 ± 3.55 | 5.66 ± 3.64 | 4.87 ± 3.51 | 5.65 ± 3.31 | 5.72 ± 3.64 | 6.45 ± 4.06 |

Abbreviation: IPAQ = International Physical Activity Questionnaire; MET = metabolic equivalent of task. Data are mean±SD.

Graphs. Distributions for PA according to Time, Level, Type of sport, and Gender. Legend for all the graphs: F = Female; M = Male; E = Elite; NE = Non-elite; I = Individual; T = Team.


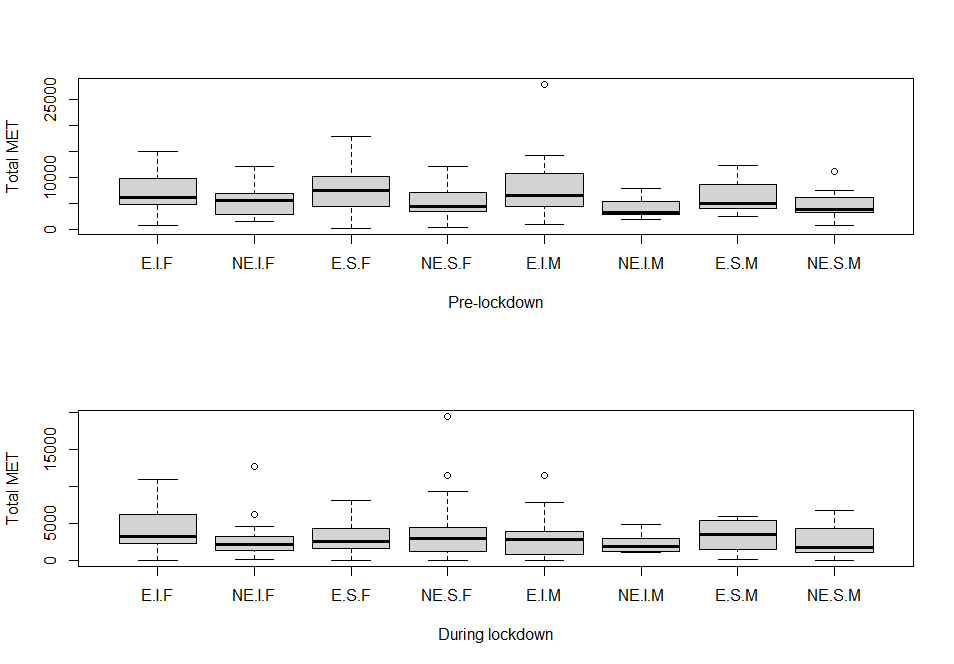

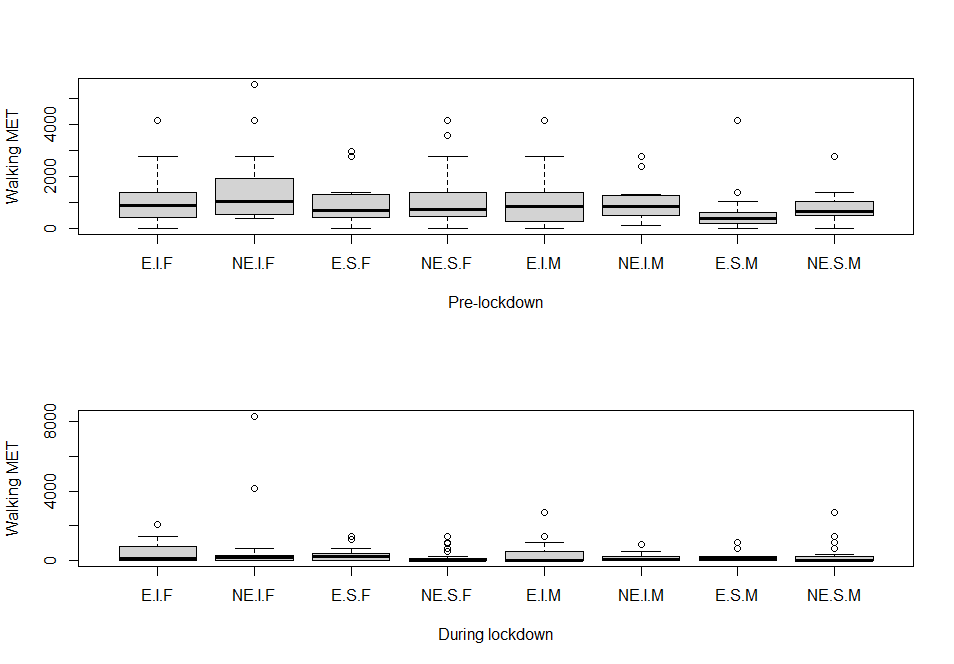

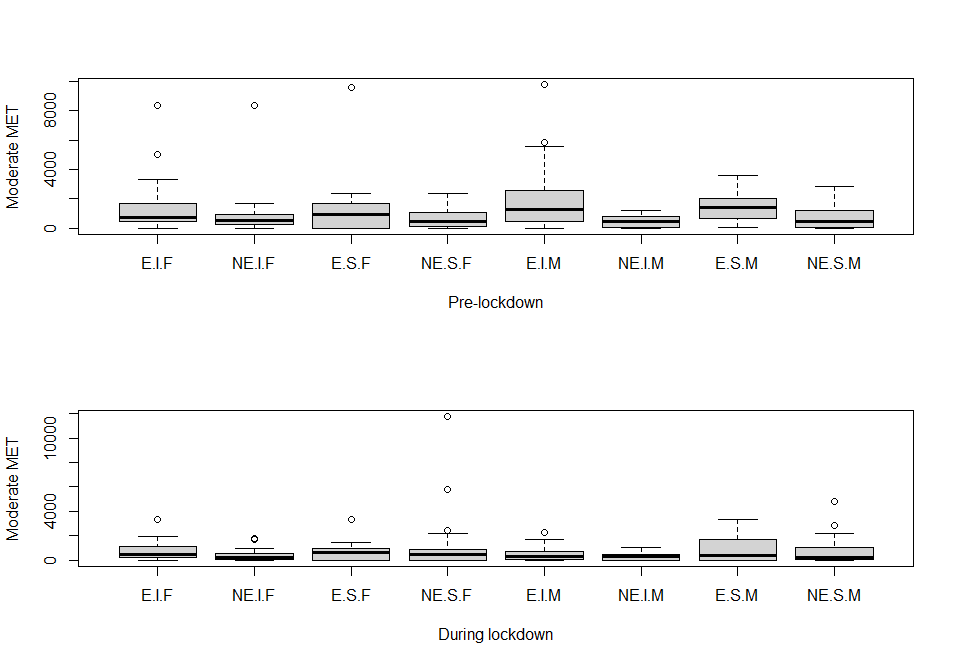

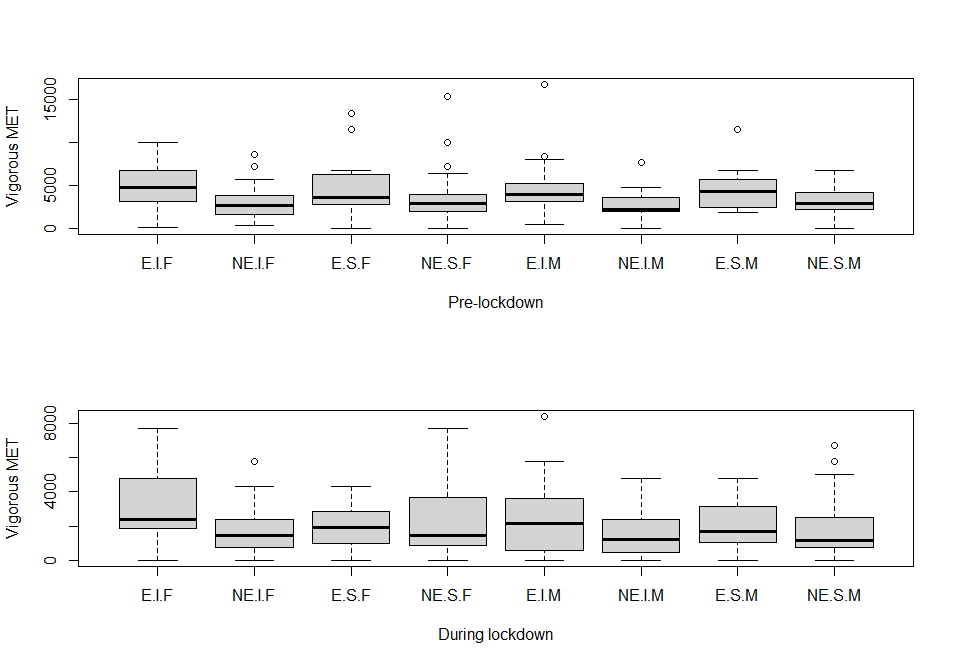


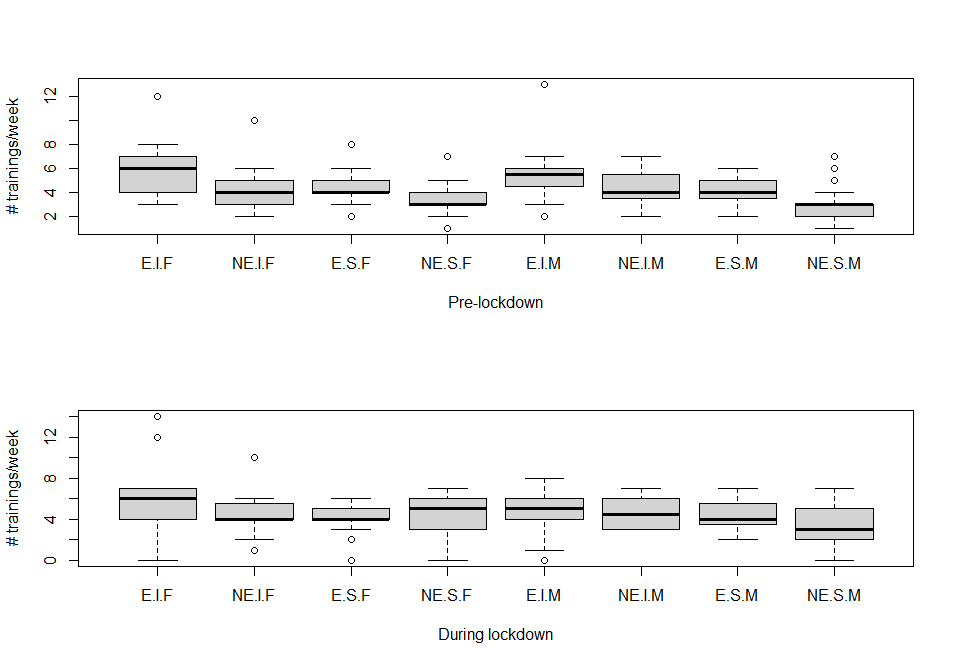

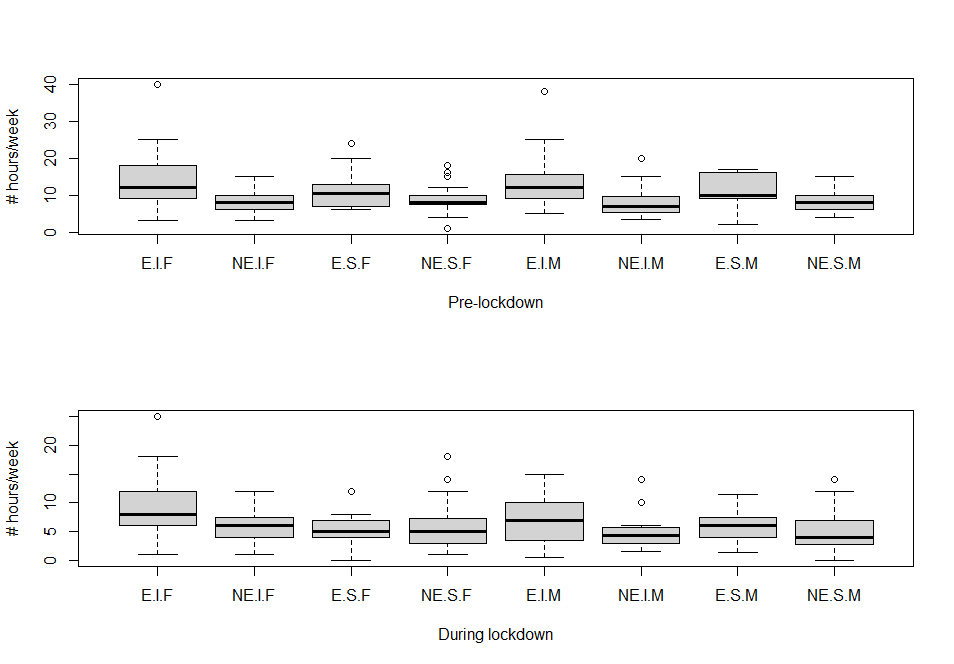

Supplement: Supplementary file 1 [file Data_Sheet_1.docx]
