# Supplementary material for: Just Do It: High Intensity Physical Activity Preserves Mental and Physical Health in Elite and Non-elite Athletes During COVID-19
Source: Front Psychol. 2021 Nov 10;12:757150. doi: 10.3389/fpsyg.2021.757150 (PMC8631504; doi:10.3389/fpsyg.2021.757150)

Supplementary File 2. Means and standard deviations of psychological variables according to Time, Level, Type of sport, and Gender.

|  | Level | Individual | | Team | | Total (individual + team) | | Total (elite + non elite) | |
| --- | --- | --- | --- | --- | --- | --- | --- | --- | --- |
|  |  | Males | Females | Males | Females | Males | Females | Males | Females |
| **Amotivation** | *Elite* | 0.52 ± 1.09 | 0.38 ± 1.1 | 1.37 ± 2.36 | 0.44 ± 1.04 | 0.87 ± 1.76 | 0.41 ± 1.06 | 0.8 ± 1.61 | 0.58 ± 1.31 |
|  | *Non-elite* | 0.83 ± 1.53 | 0.67 ± 1.57 | 0.69 ± 1.47 | 0.71 ± 1.37 | 0.72 ± 1.47 | 0.69 ± 1.45 |  |  |
| **External regulation** | *Elite* | 1.11 ± 2.17 | 0.85 ± 1.59 | 1.42 ± 1.46 | 0.67 ± 1.37 | 1.23 ± 1.9 | 0.77 ± 1.49 | 1.2 ± 2.14 | 1.06 ± 2.02 |
|  | *Non-elite* | 0.67 ± 1.5 | 0.42 ± 1.06 | 1.34 ± 2.61 | 1.79 ± 2.72 | 1.17 ± 2.38 | 1.25 ± 2.3 |  |  |
| **Introjected regulation** | *Elite* | 5.39 ± 2.69 | 6.73 ± 2.84 | 5.42 ± 2.95 | 5.44 ± 2.99 | 5.4 ± 2.76 | 6.2 ± 2.94 | 5.53 ± 2.66 | 5.81 ± 2.95 |
|  | *Non-elite* | 5.33 ± 3.37 | 5.19 ± 3.45 | 5.76 ± 2.28 | 5.77 ± 2.58 | 5.65 ± 2.58 | 5.54 ± 2.95 |  |  |
| **Identified regulation** | *Elite* | 13.41 ± 2.27 | 14.38 ± 1.5 | 12.68 ± 2.77 | 13.83 ± 1.95 | 13.11 ± 2.49 | 14.16 ± 1.7 | 12.82 ± 2.67 | 13.73 ± 2.03 |
|  | *Non-elite* | 13.75 ± 2.49 | 13.92 ± 2.24 | 12.11 ± 2.87 | 13.11 ± 2.12 | 12.53 ± 2.84 | 13.44 ± 2.19 |  |  |
| **Intrinsic regulation** | *Elite* | 14.43 ± 1.89 | 14.58 ± 1.7 | 12.89 ± 2.45 | 13.67 ± 2.17 | 13.81 ± 2.24 | 14.2 ± 1.94 | 13.05 ± 3.07 | 13.54 ± 2.31 |
|  | *Non-elite* | 13.92 ± 3.92 | 14.08 ± 1.8 | 11.74 ± 3.35 | 12.44 ± 2.61 | 12.3 ± 3.59 | 13.08 ± 2.45 |  |  |
| **DASS-21 total score** | *Elite* | 13.21 ± 11.81 | 21.27 ± 12.42 | 13.63 ± 5.85 | 15.06 ± 8.91 | 13.38 ± 9.76 | 18.73 ± 11.43 | 13.21 ± 9.24 | 18.45 ± 11.2 |
|  | *Non-elite* | 12.42 ± 8.89 | 16.89 ± 10.35 | 13.26 ± 8.89 | 19.21 ± 11.68 | 13.04 ± 8.8 | 18.26 ± 11.13 |  |  |
| **IUS-R total score** | *Elite* | 30.14 ± 7.49 | 30.5 ± 8.67 | 27.32 ± 4.73 | 27.61 ± 7.64 | 29 ± 6.61 | 29.32 ± 8.3 | 28.4 ± 6.97 | 29.02 ± 8.59 |
|  | *Non-elite* | 25.67 ± 6.01 | 27.81 ± 8.17 | 28.54 ± 7.67 | 29.51 ± 9.32 | 27.81 ± 7.33 | 28.82 ± 8.84 |  |  |
| **Physical component** | *Elite* | 55.27 ± 6.95 | 55.25 ± 7.46 | 54.98 ± 3.29 | 56.1 ± 4.8 | 55.14 ± 5.55 | 55.58 ± 6.5 | 55.3 ± 4.88 | 55.34 ± 5.62 |
|  | *Non-elite* | 54.25 ± 6.47 | 54.56 ± 5.29 | 55.79 ± 3.4 | 55.56 ± 4.69 | 55.48 ± 4.12 | 55.16 ± 4.92 |  |  |
| **Mental component** | *Elite* | 43.31 ± 12.17 | 37.16 ± 12.32 | 43.47 ± 11.46 | 38.4 ± 9.35 | 43.38 ± 11.7 | 37.65 ± 11.14 | 42.18 ± 11.08 | 37.61 ± 10.91 |
|  | *Non-elite* | 37.72 ± 14.62 | 38.94 ± 12.15 | 41.67 ± 9.22 | 36.69 ± 9.94 | 40.88 ± 10.39 | 37.59 ± 10.83 |  |  |
| **BD total score** | *Elite* | 24.61 ± 8.61 | 33.19 ± 10.95 | 25.79 ± 9.62 | 36.17 ± 11.92 | 25.09 ± 8.95 | 34.41 ± 11.32 | 25.13 ± 8.79 | 33.32 ± 10.81 |
|  | *Non-elite* | 22.92 ± 9.3 | 28.74 ± 10.04 | 25.94 ± 8.51 | 35.26 ± 10.05 | 25.17 ± 8.72 | 32.59 ± 10.48 |  |  |

Abbreviation: BD = Body Dissatisfaction; DASS-21 = Depression Anxiety Stress Scales-21; IUS-R = Intolerance of Uncertainty Scale-Revised. Data are mean±SD.

Graphs. Distribution of scores for each psychological variable according to Level of sport, Type of sport, and Gender. Legend for all the graphs: F = Female; M = Male; E = Elite; NE = Non-elite; I = Individual; T = Team.


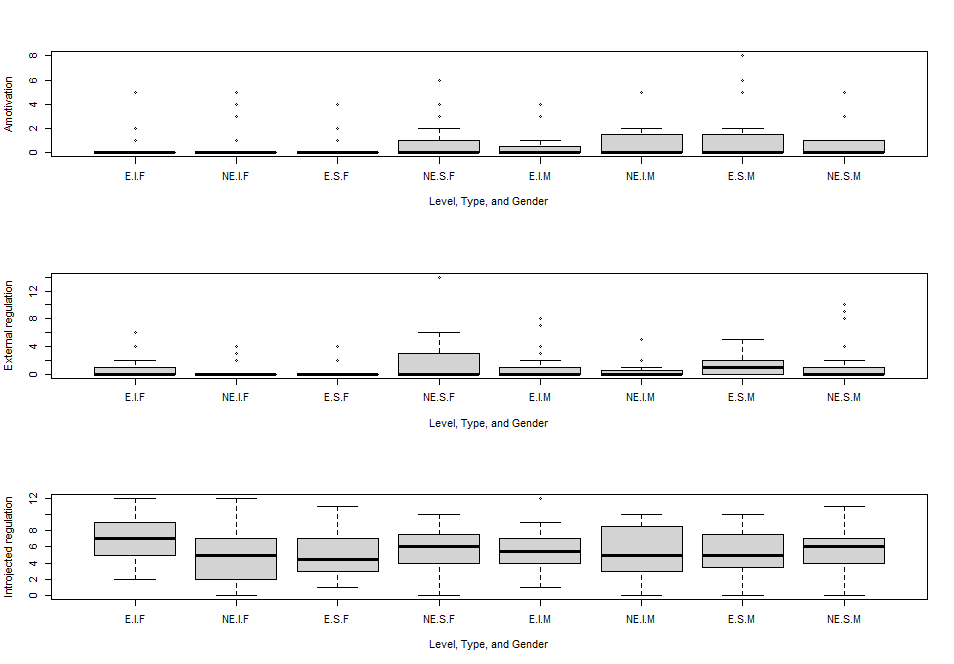

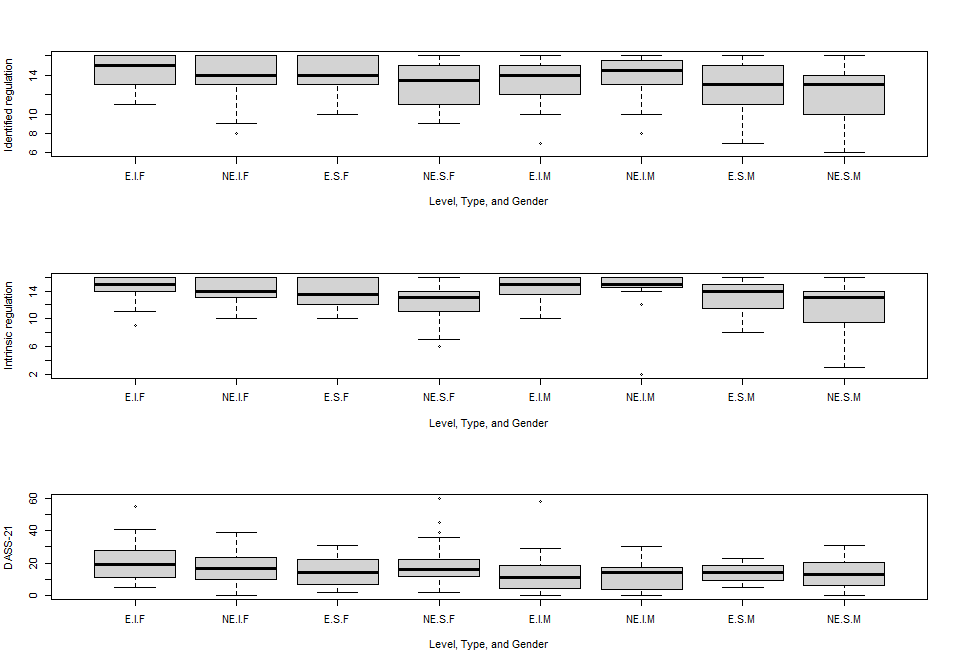


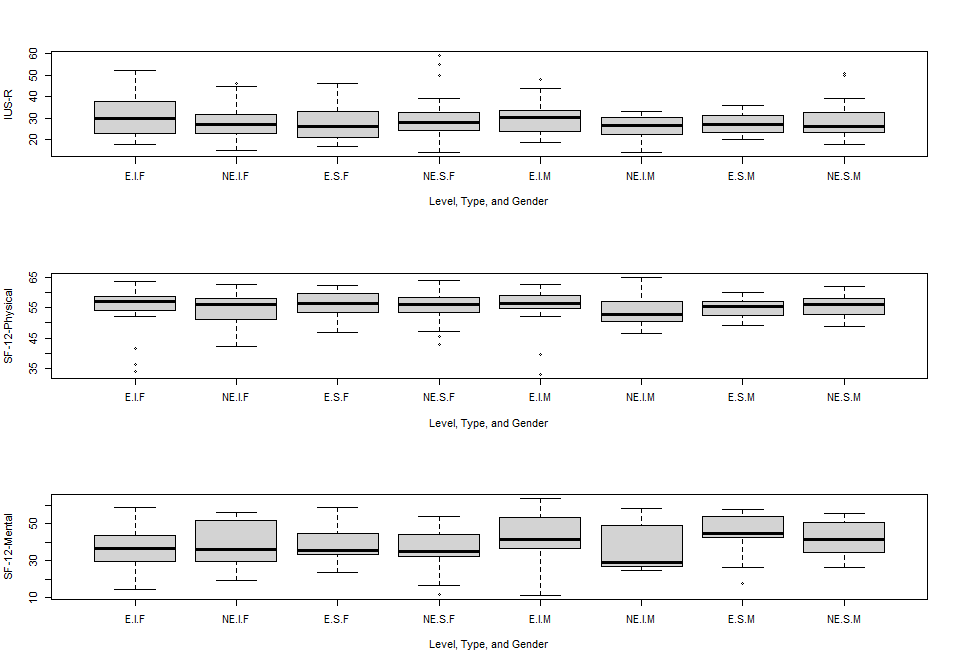

Supplement: Supplementary file 2 [file Data_Sheet_2.docx]
